# Nanoparticles for antiviral nucleotide analogs: approaches and analytical challenges

**DOI:** 10.1128/jvi.01758-25

**Published:** 2026-07-30

**Authors:** Scott M. Clifford, Matthew J. Edwards, Woravimol Krittaphol, Greg F. Walker, Lawrence D. Harris, Vernon K. Ward

**Affiliations:** 1 Department of Microbiology and Immunology, Faculty of Biomedical Sciences, University of Otago 626306, Dunedin, New Zealand; 2 School of Pharmacy and Pharmacology, University of Otago 2495, Dunedin, New Zealand; 3 The Ferrier Research Institute, Victoria University of Wellington 8491, Wellington, New Zealand; Indiana University Bloomington, Bloomington, Indiana, USA

**Keywords:** nanoparticles, antiviral agents, nucleotide analogs, drug delivery

## Abstract

Nucleoside and nucleotide analogs are an impactful class of antivirals, yet their efficacy is often limited by inefficient intracellular accumulation and enzymatic activation. Many analogs are administered as nucleoside prodrugs, dependent on host or viral kinases for sequential phosphorylation, with the first monophosphate-forming step widely considered rate-limiting. Medicinal chemistry strategies such as utilizing masked-phosphate prodrugs may improve uptake and activation, but require alternative metabolic processing to generate the active triphosphate. Nanoparticle delivery provides an alternative or complementary approach by directly encapsulating phosphorylated nucleotide analogs. Nanoparticles made from materials including lipids, polymers, dendrimers, or polysaccharides can protect phosphorylated drugs from degradation, improve cellular uptake, and enable controlled or tissue-specific release. This review provides a brief context and overview of recent advances in nanoparticle-mediated delivery of phosphorylated antiviral nucleotides, drawing on parallels from oncology, and evaluates the technological advances and limitations influencing their continued development as antiviral carriers.

## INTRODUCTION

Viral infections are a major economic and epidemiological burden on modern society, responsible for millions of deaths each year, and often have a disproportionate burden on at-risk and vulnerable populations. Chronic infections caused by hepatitis viruses and human immunodeficiency virus (HIV) result in 1.3 million and 0.6 million annual deaths, respectively (1, 2). Acute respiratory viruses, including influenza virus, respiratory syncytial virus, and severe acute respiratory syndrome coronavirus 2 (SARS-CoV-2), are among the most common global viral infections (3). Influenza virus infections number approximately 1 billion each year, causing between 0.29 million and 0.65 million deaths, with the majority in populations 65 or older (3). The COVID-19 pandemic was the most consequential global health event since the 1918 H1N1 influenza virus pandemic. According to the World Health Organization, as of September 2025, there have been 7.1 million COVID-19-related deaths (https://data.who.int/dashboards/covid19/deaths?n=o), with age being the strongest risk factor for severe COVID-19 and death (3).

Trends such as urbanization and climate change have increased the likelihood of a new viral pandemic emerging in the foreseeable future (4). Although complete vaccination would be ideal, effective vaccine development is not always possible due to factors including viral genetic diversity, rapid mutation, and complex host immune responses (5). For some viruses, a lack of suitable cell culture or animal models and reduced fiscal incentives limit vaccine development (5). Furthermore, approved vaccines may be more effective at reducing severe disease than preventing transmission (6). In parallel, increasing vaccine hesitancy poses an additional threat (7). As such, the absence or ineffectiveness of vaccination necessitates the continued use of antivirals.

Antivirals can be divided into two broad categories: those that target host-cell factors required for the viral lifecycle and those that directly inhibit viral proteins (8). Although host-targeting strategies have shown some success, the increased risk of toxicity limits their development and clinical adoption, meaning direct-acting antivirals are more widely utilized (9, 10). These inhibitors typically target key viral enzymes, such as proteases, helicases or polymerases, or block viral attachment and entry (10). Recent examples include nirmatrelvir, a SARS-CoV-2 protease inhibitor, and the hepatitis C virus (HCV) nucleotide analog sofosbuvir (SOF) (11, 12).

Among direct-acting antivirals, nucleoside and nucleotide analogs targeting DNA/RNA polymerases, reverse transcriptase, and other replication complexes have a longstanding track record in treating viral infections. In 1963, a nucleoside analog prodrug called idoxuridine became the first Food and Drug Administration (FDA)-approved antiviral drug and was effective against herpes simplex virus type 1 (13). In the years since, over 30 nucleoside/tide analogs have been approved for clinical use, making them one of the most established and widely utilized classes of antiviral therapeutics (14). However, the continued emergence of viral threats and antiviral resistance demands ongoing antiviral discovery and development (15). Given their proven clinical relevance across numerous virus families and ongoing demand, a critical evaluation of strategies to enhance nucleotide analog efficacy and delivery is warranted.

**Fig 1:**
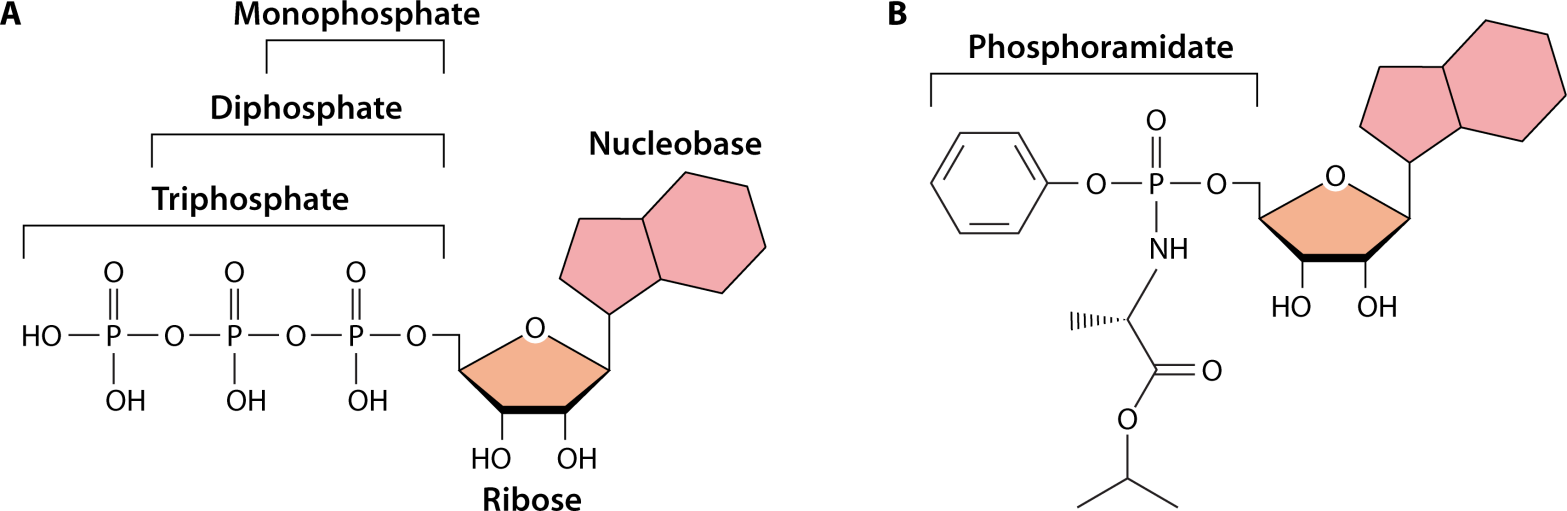
Nucleotide structure. (**A**) Schematic of a purine nucleotide triphosphate (NTP). Purine nucleobase, ribose, and phosphate groups are indicated. (**B**) Schematic of ProTide phosphoramidate modification. Figure created with BioRender.com.

Nucleotides consist of a purine or pyrimidine base, a ribose, and one or more negatively charged phosphates (Fig. 1). Due to the polar nature of the phosphate groups, active di- or triphosphate nucleotides are generally cell-impermeable, which restricts their cellular uptake (16, 17).

**Fig 2:**
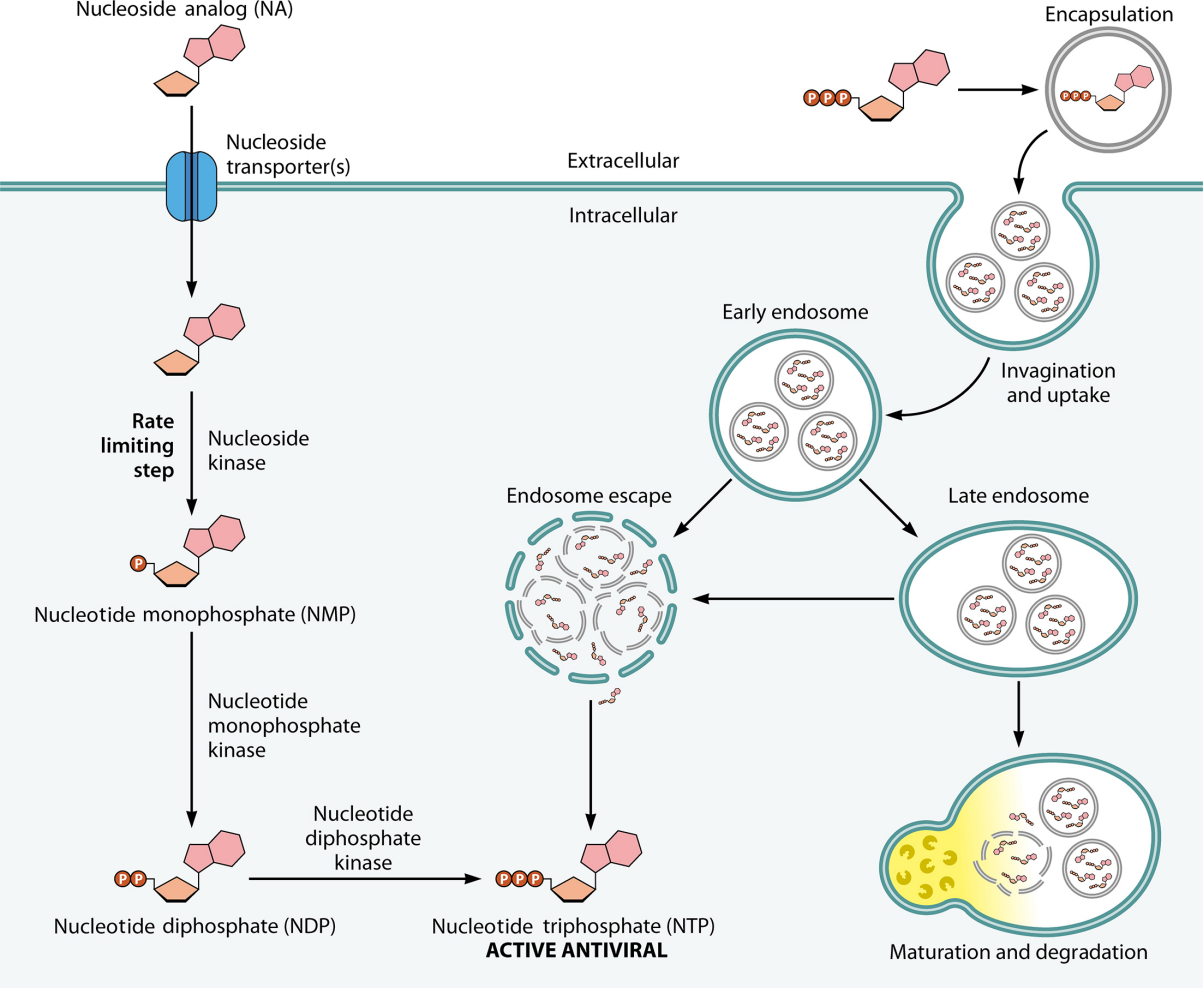
Schematic comparing delivery and activation of nucleoside analogs and nanoparticle-encapsulated nucleotide triphosphates. Figure created with BioRender.com.

Nucleotide drugs are often administered as non-phosphorylated nucleoside analogs, thereby facilitating effective influx through nucleoside transporter channels without the need for specialized delivery systems (Fig. 2) (13). Nucleosides require intracellular activation by host or viral kinases to generate their active di- or triphosphate species, with the first monophosphate-forming step widely considered rate-limiting (18–20). This process can be inefficient due to variability in kinase activity and nucleoside transport (21). Hydrophilic analogs typically show limited accumulation in the central nervous system because they exhibit variable active uptake and poor passive membrane diffusion across the restrictive blood-brain barrier (22). Consequently, effective treatment of neurotropic viruses remains a major challenge for the class. Medicinal chemistry strategies, such as utilizing masked-phosphate prodrugs, can increase lipophilicity and improve passive cellular entry, enabling the formation of nucleotide monophosphate (NMP) while bypassing the rate-limiting first phosphorylation (12, 17, 23, 24). However, masked-phosphate modifications can reduce intracellular delivery and add additional metabolic processing to generate the active phosphorylated forms (25). For example, phosphoramidate modified prodrugs (ProTides) of 2′-fluoro derivatives of pentylphenyl bicyclic nucleoside analog were not more effective than parent compounds at inhibiting varicella-zoster virus *in vitro*. This effect was attributed to inefficient release of NMP or failure to phosphorylate to the active form (26). Collectively, these limitations highlight a key gap in the direct delivery of phosphorylated nucleotide analogs.

Nanoparticle delivery of phosphorylated nucleotide analogs may improve intracellular accumulation while bypassing inefficient enzymatic phosphorylation steps that can limit nucleoside antiviral activity. This review discusses nanoparticles as carriers for antiviral nucleotide analogs, while also considering masked-phosphate nucleotides when drug lipophilicity facilitates encapsulation. Nanoparticle design, fabrication, and physicochemical properties, such as size and surface charge, are examined alongside challenges in quantifying the percentage of initial drug successfully encapsulated in the nanoparticle (encapsulation efficiency, EE) and the amount of drug per carrier mass (drug loading, DL) (27). These challenges are considered alongside *in vitro* and *in vivo* data to highlight a pathway to improved reproducibility and downstream clinical viability for antiviral nanoparticles, aligning with the growing adoption of other nanomedicines.

The success of nanomedicine during the SARS-CoV-2 pandemic has renewed interest in the nanoparticle-based delivery of nucleic acid, peptide, and small molecule antivirals. Since 2025, over 5 billion doses of Pfizer-BioNTech COVID-19 lipid nanoparticle (LNP)-mRNA vaccine have been delivered, marking one of the most widely adopted nanomedicines in human history and paving the way for continued innovation (https://www.pfizer.com/news/press-release/press-release-detail/pfizer-and-biontech-announce-topline-data-demonstrating). In addition to lipid systems, nanoparticles composed of polymers, dendrimers, and inorganic metals offer a range of developmental and therapeutic attributes, including protection from degradation and potentially improved delivery when compared to unencapsulated nucleoside/tide analogs (28–31).

**Fig 3:**
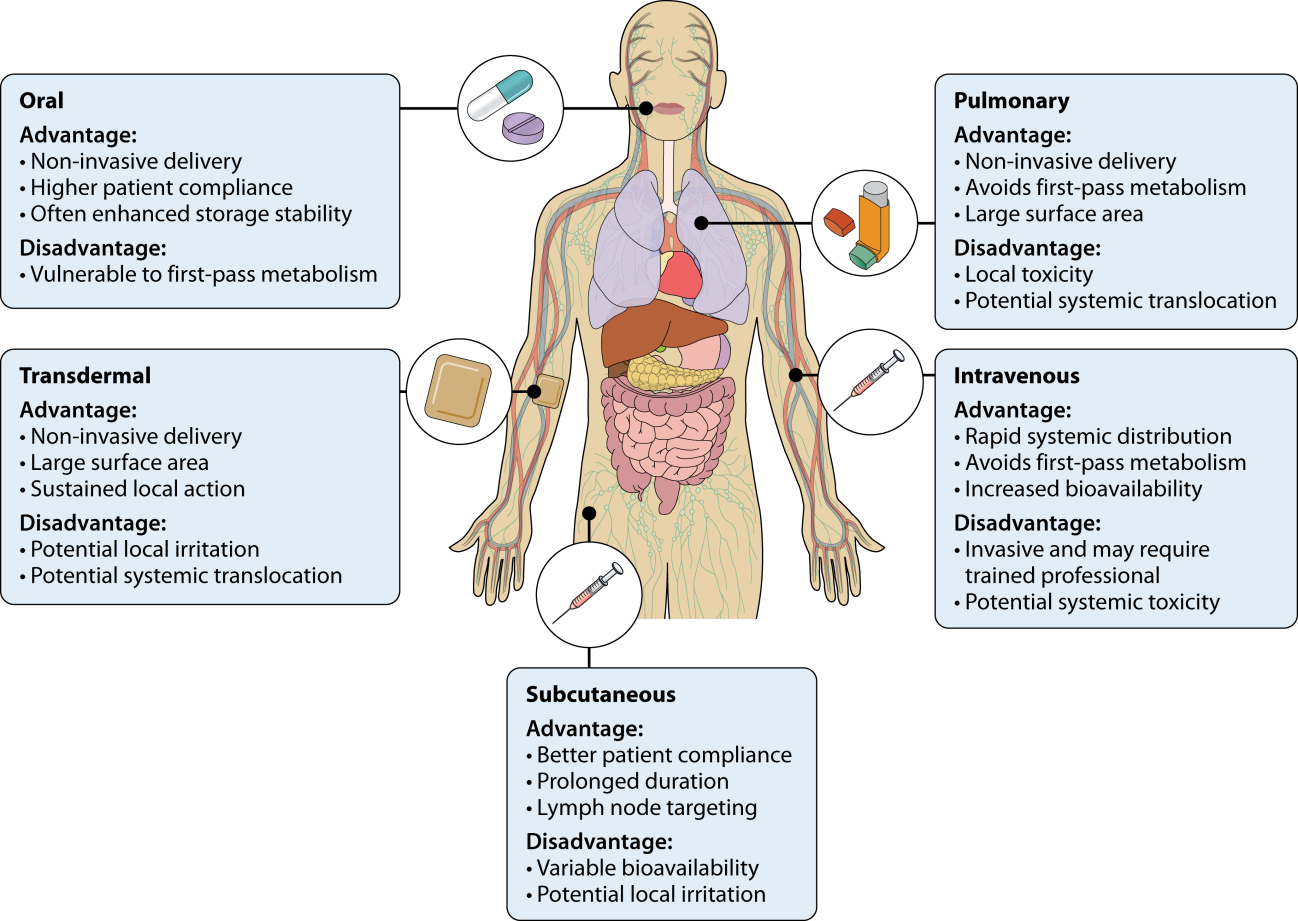
Overview of administration routes for nanoparticles. Adapted from Yildirimer et al. (38), previously published under a Creative Commons license (38). Figure created with BioRender.com.

Nanoparticles can be formulated for specific routes of administration (Fig. 3), enabling more direct targeting of the site of viral infection or disease pathology (32). For respiratory viruses, inhaled or intranasally administered nanoparticles can deliver antivirals directly to infected respiratory cells, circumventing first-pass metabolism (33). Efficient deep-lung deposition is generally achieved using aerosol droplets, nanoparticle aggregates, or microparticle carriers with aerodynamic diameters between 1 and 5 µm (34). Following pulmonary deposition, nanoparticles under 1,000 nm are more likely to traverse the mucosal barrier and avoid mucociliary or phagocytic clearance (35). Nanoparticles smaller than 100 nm can exhibit increased translocation into the circulatory or lymphatic system (35). Particle charge, hydrophobicity, and other physicochemical properties also significantly impact the intrapulmonary fate of inhaled nanoparticles (34). Although pulmonary administration facilitates greater accumulation in infected lung tissue, viral replication in other organs should also be considered, particularly for viruses such as SARS-CoV-2, which replicate at multiple sites in the body (36, 37).

Intravenous administration is frequently used in emergencies as it enables rapid systemic distribution and bypasses first-pass metabolism (32). Within the blood, particle size, surface chemistry, and morphology impact circulation half-life, clearance by the mononuclear phagocyte system (MPS), and tissue distribution (32). Nanoparticles with near-neutral charge often demonstrate improved circulation time. Highly positive or negatively charged nanoparticles are more prone to protein absorption and rapid clearance from circulation by resident MPS cells, such as Kupffer cells and splenic macrophages, thereby increasing accumulation in the liver and spleen (32, 39).

Nanoparticles with solid lipid or polymer cores are well-suited for oral formulations because they protect antiviral cargo from the harsh acidic environment of the gastrointestinal (GI) tract (40). Neutral and/or hydrophilic nanoparticles are more likely to avoid entrapment within the GI mucosal layer (41). If a nanoparticle successfully traverses this protective barrier, it can be endocytosed by small intestinal epithelial cells such as enterocytes, potentially enabling transport to the portal vein and subsequent first-pass metabolism (42). Alternatively, nanoparticles can be optimized for uptake by specialized microfold (M) cells within the gut-associated lymphoid tissue of the ileum, which provides preferential access to the lymphatic pathway (42).

Local injection routes, such as subcutaneous, enable absorption via the lymphatic system prior to potential systemic circulation (39). Subcutaneous administration of nanoparticles between 10 and 100 nm can enhance uptake into the lymphatic system, which is particularly important for antiretroviral nanoparticles targeting HIV reservoirs, such as CD4-T cells (43). Additionally, subcutaneous formulations can improve pharmacokinetics, including plasma drug half-life. A subcutaneous nanoparticle formulation containing the anti-HIV drugs atazanavir, darunavir, and tenofovir (TFV) resulted in sustained plasma drug concentrations for 1 week, highlighting the potential of a long-acting once-weekly formulation (44).

Following administration, nanoparticles enter cells primarily via two mechanisms: either direct fusion with the plasma membrane or endocytic uptake (Fig. 2), with the latter being the predominant route of cellular internalization (45, 46). Endocytosis encompasses diverse uptake pathways in which interactions between nanoparticles and the plasma membrane drive invagination and vesicle formation (47). This process includes pinocytosis for small particles and phagocytosis for larger particles, which is a function of specialized phagocytic cells such as macrophages (48).

Following cellular internalization, nanoparticles are generally delivered to the early endosome through a Rab5a/EEA1-dependent mechanism regardless of the internalization pathway (49). From the early endosome, internalized nanoparticles may undergo several distinct trafficking fates (46). These include recycling back to the plasma membrane for cargo release out of the cell, retrograde transport to the endoplasmic reticulum—Golgi network, or retention in the endosomal lumen. The early endosome can then progress and mature into a late endosome, ultimately forming multivesicular bodies (MVBs) (46). Subsequent fusion of the late endosome/MVB with lysosomes leads to acidification and enzymatic degradation of the retained nanoparticles and a breakdown of associated cargo (46). Therefore, escape from the endosome before degradation represents a major bottleneck for functional delivery of nanoparticle-delivered antivirals (50). For most lipid and polymeric systems, less than 5% of internalized cargo successfully reaches the cytosol, as the majority is recycled outside of the cell or degraded in subsequent lysosomes (51, 52).

Precise mechanisms for endosomal escape require continued characterization. Carrier physicochemical properties, cargo, target cell type, and the route of endocytosed particles are known to impact the efficiency of cytosolic cargo release (51). Leading theories include the proton sponge effect, membrane fusion/destabilization, and mechanical force. The proton sponge effect has been attributed to the buffering capacity of certain polycationic polymers or lipids, which activate proton influx via the endosomal ATPase (53). The influx of coupled chloride ions and water molecules causes endosomal swelling and rupture. Destabilization and/or fusion-mediated escape result from interactions between nanoparticles and endosomal membranes (54). For example, ionizable lipids are thought to facilitate escape by undergoing pH-dependent protonation and interacting with anionic lipids within the endosomal membrane (51). This interaction induces non-bilayer structural changes, damaging the membrane and releasing cargo (51). The inclusion of fusogenic proteins or peptides on the nanoparticle surface can also cause destabilization or fusion, thereby enhancing endosomal escape (55–57).

## LIPID NANOPARTICLES

### Liposomes

First investigated in the 1960s, liposomes are one of the earliest and most frequently described drug-carrying nanoparticles (58). Liposomes are self-assembled phospholipid-based drug vesicles that form uni-lamellar bilayers and/or a series of multilamellar bilayers enclosing an aqueous core, capable of encapsulating both hydrophobic and hydrophilic cargo (59, 60). Hydrophilic drugs such as nucleotide analogs have lower membrane permeability, improving their retention within the liposome (59). To enhance stability and circulation times, many liposomes are now formulated with hydrophilic polymers such as polyethylene glycol (PEG) (61). Liposomes allow improved delivery, site targeting, and protection of drugs from both degradation and clearance (59–61). This has translated into sustained clinical adoption over several decades, with at least 13 liposomal formulations approved for use in the United States and/or European Union (60).

**Fig 4:**
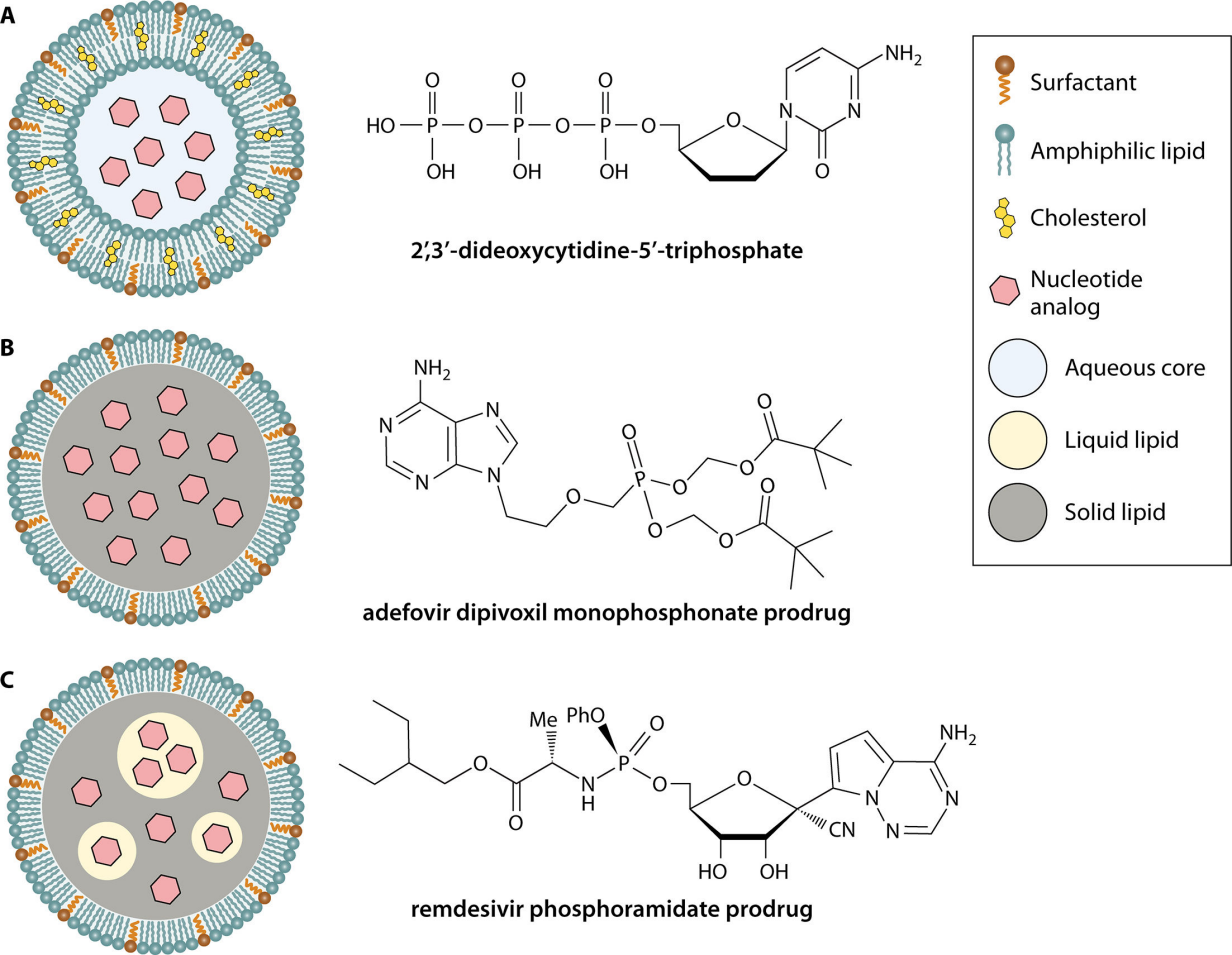
Schematic of lipid-based delivery of antiviral nucleotides. (**A**) Liposomal encapsulation of the nucleotide 2′,3′-dideoxycytidine-5′-triphosphate. (**B**) Solid lipid nanoparticle (SLN) formulation containing adefovir dipivoxil monophosphonate prodrug. (**C**) Nanostructured lipid carrier (NLC) encapsulating remdesivir phosphoramidate prodrug. Representative chemical structures are shown alongside their respective carrier. Figure created with BioRender.com.

Thoroughly discussed elsewhere, early antiviral liposome progress primarily utilized thin film extrusion methods to encapsulate anti-HIV NTPs such as 2′,3′-dideoxycytidine-5′-triphosphate (ddCTP) and 3′-azido-3′-deoxythymidine (AZT) with the goal of reducing toxicity and improving delivery to phagocytotic cells, key reservoirs of HIV (Fig. 4A) (62, 63). *In vitro* and *in vivo* murine data indicated that these liposomal formulations were capable of improving delivery, retention, and reducing toxicity (64–66). However, Szebeni et al. found that despite having low nM anti-HIV activity, ddCTP liposomes were approximately five times less potent than the ddC nucleoside prodrug (66). Encapsulation of hydrophilic and highly polar analogs such as ddCTP and AZT in liposomes is generally limited, with EE typically between 9% and 26%. Despite this, foundational studies provided extensive descriptions of methodology and analytical validation, providing a framework for future progress (64–66).

In response to the SARS-CoV-2 pandemic, liposomes were also investigated as a delivery strategy for remdesivir (RDV), a lipophilic phosphoramidate (ProTide) prodrug form of an adenosine nucleoside analog. RDV is limited by low aqueous solubility and poor accumulation in lung tissue following intravenous administration, reducing formation of active triphosphate at the site of infection (67). Therefore, a similar thin-film hydration method was utilized to make liposomes consisting of 1,2-dipalmitoyl-sn-glycero-3-phosphocholine, cholesterol, and 1,2-distearoyl-sn-glycero-3-phosphoethanolamine-N-[methoxy(polyethylene glycol)-2000] (DSPE-PEG 2000) loaded with RDV (68). These liposomes had substantially higher EE and DL when measured via ultraviolet (UV) spectrophotometry, reaching 89.1% and 11.1%, respectively. Others have reported RDV liposomes with EE between 57.2% and 99.8%, highlighting a broad range, likely influenced by formulation methodology and analysis (69, 70). When inhaled, the RDV-liposome formulation resulted in a 100-fold increase in active NTP accumulation in murine lung when compared with an intravenous cyclodextrin-based RDV solution, as measured by liquid chromatography-tandem mass spectrometry. More recently, Mendes et al. showed that intranasal liposomal RDV significantly reduced SARS-CoV-2 viral load in lung and brain tissue and led to complete survival in infected mice, confirming enhanced antiviral efficacy *in vivo* (69).

## SOLID LIPID NANOPARTICLES

SLNs have become a popular delivery system because they offer enhanced stability over conventional lipid carriers and can be manufactured using flexible, scalable lipid-based technologies (71). SLNs consist of a highly organized dense lipid-drug matrix core and surfactants. This solid lipid core allows for long-term storage in aqueous solution, unlike their liposome predecessors (72). Although capable of carrying both hydrophobic and hydrophilic drugs, water-soluble drugs tend to partition into the aqueous phase during manufacturing (71, 73). SLN formulations with water-soluble nucleotide analogs, such as the acyclic adenosine monophosphate analog TFV, have demonstrated <10% EE (74). Consequently, SLN-based systems are more feasible for lipophilic antiviral nucleotides as they preferentially associate with the lipid phase. For example, Zhang et al. encapsulated adefovir dipivoxil (ADV), a lipophilic acyclic adenosine monophosphonate prodrug, with the aim of treating hepatitis B virus (HBV) (Fig. 4B) (75, 76). This study utilized monostearin as the lipid matrix and prepared SLNs using a previously published solvent diffusion method (77). Particles had a mean size of 389.4 ± 166.5 nm with a polydispersity index (PDI) of 0.37, indicating moderate heterogeneity was achieved. The 15.3% EE and 3.1% DL were determined using UV measurement of the supernatant at 260 nm wavelength following centrifugation. ADV-loaded SLNs significantly reduced HBV DNA levels compared with ADV in solution after both 2 and 5 days of incubation in HepG2.2.15 human hepatoma cells stably transfected with the HBV genome (76). In a separate study, SLNs encapsulating tenofovir alafenamide (TAF) achieved substantially higher EE of 96.1%, despite having a similar octanol-water partition coefficient to ADV (TAF logP 1.9; ADV logP 1.8) (PubChem). This indicates that formulation and analysis methodology have a larger impact than lipophilicity alone. In contrast to the ADV method, TAF SLNs were prepared using melt emulsification, allowing the drug to dissolve within the molten lipid and become entrapped during recrystallization (78).

## NANOSTRUCTURED LIPID CARRIERS

In 1999, NLCs were developed to overcome the limited DL capacity of SLNs (72). These second-generation colloidal nanoparticles closely resemble the outer structure of SLNs but are differentiated by their disorganized lipid-liquid and solid lipid core (72). This complex matrix facilitates higher DL capacity and is reported to further enhance long-term stability and drug retention (72).

Recently, Jeon et al. utilized NLCs to encapsulate and deliver RDV phosphoramidate prodrug (Fig. 4C) (29). NLC-RDV were prepared by a hot-melt method in which glyceryl monostearate, glyceryl caprylate/caprate, and RDV were heated at 80°C and emulsified with a surfactant-containing aqueous phase, followed by homogenization and sonication. The size, PDI, and zeta potential of the resultant NLC were analyzed using electrophoretic laser scattering and were 143.0 ± 0.6 nm, 0.196, and −6.8 ± 0.6 mV, respectively. Transmission electron microscopy images provided additional evidence for both the size and expected spherical shape of the NLC. EE was determined indirectly by separating unencapsulated RDV by centrifugation and quantifying it with ultraperformance liquid chromatography-tandem mass spectrometry (UPLC-MS/MS). The EE was calculated from the difference between the total amount of RDV added and the amount of unencapsulated RDV measured via UPLC-MS/MS.

Indirect measures of EE are widely accepted, but they may not account for any nucleotides lost or degraded during heating, homogenization, sonication, or filtration. The Jeon et al. study reported 99.9% EE, indicating almost all of the RDV was encapsulated. An *in vitro* release experiment demonstrated that 24 h cumulative release values for RDV and NLC-RDV were 91.1% ± 6.8% and 37.2% ± 4.2%, respectively. At the final 48 h time point, 63.8% ± 5.7% of NLC-RDV was released. This was interpreted as NLC-RDV showing sustained release properties. However, as EE was measured indirectly, an accurate determination of release may be challenging.

The antiviral activity of the NLC-RDV was comprehensively tested against SARS-CoV-2 and variants in Vero E6 cells. In a CellTiter 96 colorimetric cytopathic effect assay, NLC-RDV demonstrated significantly better potency than free RDV, with half maximal effective concentration (EC_50_) of 0.16 µM and 1.6 µM, respectively. Enhanced potency was also observed for Alpha (B.1.1.7), Beta (B.1.351), and Delta (B.1.617.2) variants. Consistent with these findings, viral plaque assay and reverse transcription-quantitative polymerase chain reaction (RT-qPCR) demonstrated significantly greater suppression of plaque formation and intracellular viral RNA, respectively. Inclusion of unloaded NLC controls confirmed that antiviral effects were due to the release of encapsulated drug rather than inherent properties of the carrier (29).

## POLYMERIC NANOPARTICLES

Poly(lactic-co-glycolic acid) (PLGA) nanoparticles are among the most widely studied polymeric carriers, valued for their biocompatibility and biodegradability (79). PLGA nanoparticles and microparticles have a long-standing track record of FDA approval starting in 1989, with at least 19 FDA-approved PLGA formulations, including indications ranging from prostatic cancer, schizophrenia, bipolar disorder, and type 2 diabetes mellitus (79–81). Many PLGA nanoparticles are formulated by emulsion-solvent evaporation or nanoprecipitation methods, often requiring a substantial amount of drug (82). Such a strategy was utilized to encapsulate the adenosine monophosphate analog tenofovir disoproxil fumarate (TDF), whereby 17 conditions were trialed, yielding optimized TDF-loaded nanoparticles with a mean size of 218 ± 3.85 nm, EE of 57.3% ± 1.6%, and a PDI of 0.23 ± 0.02 (83). This study also provided supporting evidence that the nanoparticles were stable, non-toxic *in vitro*, and efficiently delivered via the gut in male Wistar rats.

**Fig 5:**
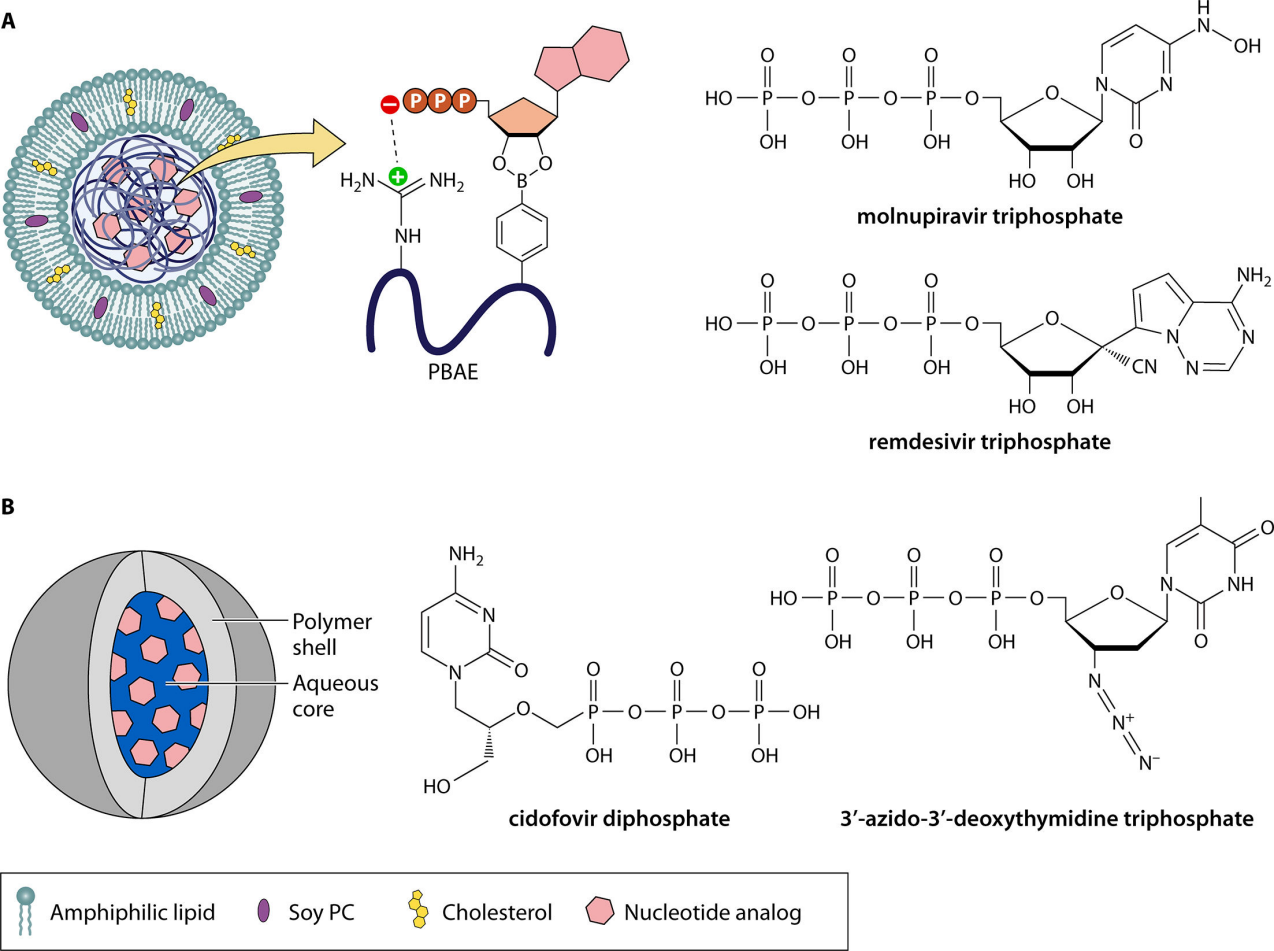
Schematic of polymer-based delivery of antiviral nucleotides. (**A**) PBAE-liposome encapsulating analogs, including molnupiravir triphosphate or remdesivir triphosphate. (**B**) Polymeric nanocapsule with cidofovir diphosphate and 3′-azido-3′-deoxythymidine triphosphate. Representative chemical structures for each example are presented adjacent to their respective carrier. Figure created with BioRender.com.

Beyond PLGA systems, polymeric nanoparticles also include affinity-engineered platforms designed to bind nucleotide analogs through multiple interactions. For example, Zhou et al. synthesized a poly(β-amino ester) polymer (PBAE) with alternating guanidine and phenylboronic acid groups (denoted as PGB), enhancing interaction between the polymer and the phosphate and ribose groups of NTPs (Fig. 5A) (84). Using a water-in-oil microemulsion system, reactive PBAE, NTP, dioleoyl-phosphatidic acid (DOPA), and Mg^2+^ were mixed under intense stirring, forming nanoparticles (84). This nanocarrier platform was used to encapsulate a range of NTP, including the triphosphate forms of molnupiravir (MTP) and remdesivir (RDV-TP), which both demonstrated an approximate size of 170 nm and a negative zeta potential of −13 mV (84). DL was reported as slightly over 50%, and EE ranged between 40% and 60% for MTP and RDV-TP, determined using high-performance liquid chromatography (HPLC).

When compared to their unencapsulated NTP control, these polymeric-NTP nanoparticles demonstrated substantially better cellular uptake across multiple cell lines. *In vitro* antiviral assays, including 50% tissue culture infectious dose (TCID_50_) and viral plaque assay, demonstrated that the polymeric-NTP nanoparticles were more effective than nucleoside prodrug and NTP forms against both porcine reproductive and respiratory virus HN07-1 and bovine enterovirus HY12 (84). Additionally, in H1N1 influenza virus-infected A549 cells, MTP-loaded nanoparticles exhibited an 8.5-fold lower 50% inhibitory concentration (IC_50_) than free molnupiravir. Consistent with these findings, in H1N1 influenza virus-infected mice, intravenous nanoparticle administration enhanced pulmonary drug accumulation, increased lung MTP retention, and protected against lung injury (84). These results highlight the advantage of polymeric delivery systems for antivirals that require intracellular phosphorylation to form active metabolites such as RDV-TP or MTP. Effective viral suppression depends on achieving sufficient intracellular concentrations early during infection. This is particularly relevant for respiratory viruses such as the influenza virus, where replication is rapid and antiviral efficacy depends on early treatment (85, 86).

## POLYMERIC NANOCAPSULES

Unlike PLGA nanoparticles, polymeric nanocapsules typically have a defined solid or liquid core and only a thin layer of surrounding polymer (87). Hillaireau et al. trialed such a system for encapsulation of AZT-TP or cidofovir diphosphate (CDV), whereby a water-in-oil microemulsion was used to incorporate nucleotides into an internal aqueous core, followed by dropwise addition of isobutylcyanoacrylate to add the polymer coating (88) (Fig. 5B). The size of these nanocapsules was 171 ± 30 nm and 169 ± 40 nm for AZT-TP and CDV, respectively. Importantly, their approach to determining EE provided a direct and quantitative measure by accounting for both encapsulated and unencapsulated nucleotide fractions. This was achieved using isotopically labeled nucleotides, allowing quantification of pellet and supernatant radioactivity by liquid scintillation following centrifugation. EE was calculated as pellet radioactivity/(pellet radioactivity + supernatant radioactivity) × 100. The EE for AZT-TP and CDV was 10%–14% and reduced sharply to approximately ≤4% when washed with phosphate-buffered saline or water, an effect hypothesized to be due to the diffusion of the nucleotides through the pores of the polymeric membrane, which did not occur with larger oligonucleotides. Although this study ultimately failed to achieve desirable EE, its method for calculating EE provided a robust base for further optimization. Subsequently, the addition of cationic polymer, poly(ethyleneimine) (PEI), to the aqueous phase increased EE to 90% (89).

## POLYSACCHARIDE AND POLYSACCHARIDE HYBRID NANOCARRIERS

Polysaccharides are naturally occurring carbohydrate compounds that play essential structural and metabolic roles across all domains of life (90). Some polysaccharides exert pharmacological activity independent of the drug they carry, with both antiviral and anticancer applications (90, 91). Through chemical or enzymatic modifications, the formation of semisynthetic polysaccharides can be generated with enhanced antiviral activity (90). Chitosan remains a widely utilized material in this category, with several studies reporting encapsulation of nucleotide analogs such as TFV, TAF, and phosphoramidate (ProTide) uridine analog SOF (92–96). Particle size and EE varied substantially between formulations, and antiviral evaluation was generally limited. For example, in an RT-qPCR assay, SOF-loaded chitosan nanoparticles reduced HCV RNA levels in infected cells at 100 µM, comparable to free drug (93). However, this concentration is several magnitudes higher than the reported <200 nM EC_50_ of SOF *in vitro*, preventing meaningful assessment of whether the nanoparticle improved potency (97).

**Fig 6:**
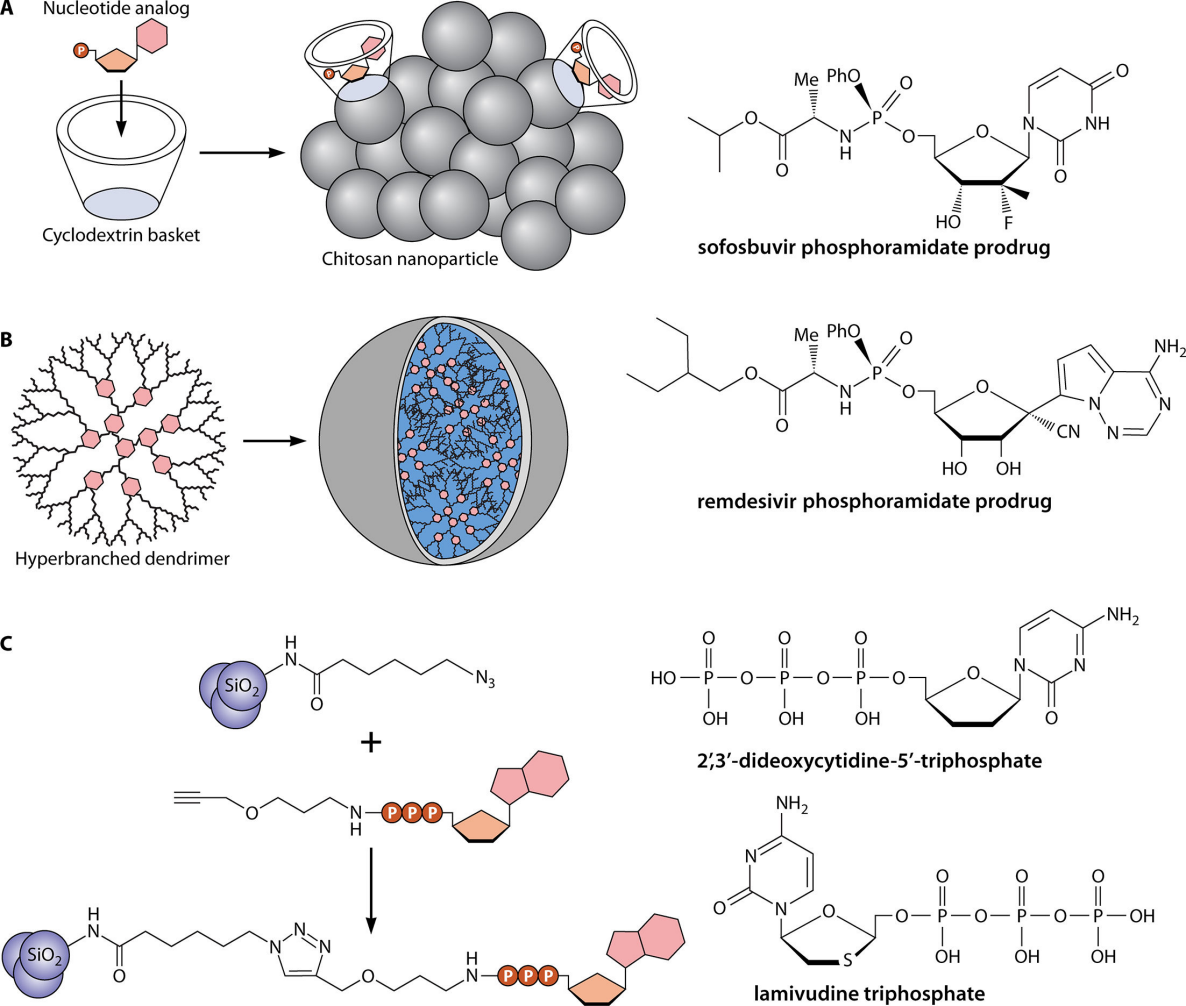
Schematic of polysaccharide, dendritic, and inorganic delivery of antiviral nucleotides. (**A**) Sofosbuvir phosphoramidate prodrug-loaded β-cyclodextrin drug baskets bound to chitosan nanoparticles. (**B**) Hyperbranched dendritic nanocarrier encapsulating remdesivir phosphoramidate prodrug. (**C**) Surface functionalized SiO_2_ nanoparticles for covalent conjugation of γ-alkynyl modified NTP, including 2′,3′-dideoxycytidine-5′-triphosphate or lamivudine triphosphate. Representative chemical structures are shown adjacent to their respective carrier systems. Figure created with BioRender.com.

Cyclic oligosaccharides such as cyclodextrin can increase the solubility and stability of antiviral nucleotide analogs, as exemplified by their use as solubilizing excipients in clinically administered RDV formulations (98, 99). Cyclodextrins form inclusion complexes in which lipophilic nucleotide analogs are accommodated within a cyclodextrin cavity, improving aqueous dispersion and potentially enabling more efficient association with nanoparticle carriers (100). The approach has been applied to hybrid carriers such as SOF-loaded β-cyclodextrin drug baskets displayed on chitosan nanoparticle surface (Fig. 6A) (101).

In a prolonged process, chitosan (Cs) nanoparticles (NPs) were formed by dropwise addition of sodium tripolyphosphate to an acidic solution of Cs and stirring for 1 day. Independently, ultrasonication was utilized to form the β-cyclodextrin-SOF (βCD-SOF) basket. The Cs and βCD-SOF solutions were mixed to form a hybrid nanocarrier by electrostatic association. The size and zeta potential for the Cs-NP were approximately 215 nm and +44 mV, respectively. The βCD-SOF formed much larger micron-scale aggregates with an approximate size of 1.4 μm and a zeta potential of −22 mV. Incorporating βCD-SOF onto Cs-NP formed a larger aggregate of material with a restored positive surface charge of +46 mV, confirming successful association of the two.

For such complex multi-component systems, determination of DL can be methodologically complex and may rely on indirect analytical approaches. In this case, loading efficiency was reported as the proportion of βCD-SOF associated with the surface of the chitosan nanoparticles, estimated from UV-vis changes rather than direct quantification of nanoparticle-bound drug. *In vitro* cytotoxicity testing was conducted across three cell lines. However, as the concentration of SOF was not clearly defined, it is difficult to directly assess the relative toxicity of the drug-loaded nanoparticle compared to the free drug.

## DENDRITIC AND HYPERBRANCHED NANOCARRIERS

Among nanocarrier systems, dendritic and hyperbranched polymers are uniquely adaptable platforms for drug delivery owing to their highly branched three-dimensional structures. Their dense array of terminal functional groups enables precise surface modification, high DL capacity, and versatile conjugation of targeting lipids (102). A study by Halevas et al. utilized an oil-in-water single emulsion/solvent method to encapsulate phosphoramidate prodrug RDV into a hyperbranched dendritic nano-scaffold (HDS) (103) (Fig. 6B). This process is enabled by the weak van der Waals forces and hydrogen bonds between the drug and the functional groups of HDS. The mean size of the RDV-HDS nanocarriers was 185.0 ± 30.5 nm, compared to the 109.0 ± 18.7 nm of the unloaded control, a difference attributed to the swelling of the nanocarrier due to entrapment with RDV (103). The 82.0% EE and 14.1% DL were determined using HPLC by directly measuring RDV in the nanoparticles (103). The authors attribute such impressive EE and DL to the large length of the PEG chain and high generation of the DLD-polymer. MTT cytotoxicity screening in lung fibroblast (MRC-5) and alveolar macrophage (NR8383) cell lines demonstrated that the unloaded nanoparticles were non-toxic at concentrations under 400 µM, and the RDV-HDS was comparable to free RDV, with 50% cytotoxicity concentration (CC_50_) of 93.60 µM and 95.11 µM in MRC-5 and NR8383, respectively.

## INORGANIC NANOPARTICLES

Inorganic nanoparticles such as silica, gold, and iron oxides have emerged as versatile carriers for antiviral nucleotide analogs owing to their high surface-to-volume ratio, chemical stability, and capacity for precise surface functionalization (104). Silicon dioxide (SiO_2_) has shown particular promise as an inorganic carrier for nucleotide triphosphates against HIV. Using a “click” style reaction, Vasilyeva et al. modified dNTP with an alkyne γ-phosphate group, enabling conjugation to azide-functionalized SiO_2_ nanoparticles (105). A subsequent Vasilyeva et al. study expanded on this work, creating ddCTP and lamivudine triphosphate (3TC-TP) conjugated SiO_2_ nanoparticles (Fig. 6C) (106).

The resulting nanoparticles had a mean size of 146.3 ± 31.6 nm when measured via NanoSight NS300 (NTA), which was substantially different from the 25–27 nm reported via small-angle X-ray scattering, an effect attributed to the propensity of SiO_2_ nanoparticles to aggregate (106). The zeta potential for the SiO_2_-3TC-TP and SiO_2_-ddCTP was −9.6 and −6.2 mV (106). Drug concentration was calculated by dissolving the SiO_2_ conjugates in 0.2 M NaOH and measuring the UV absorption. Drug concentration was presented as 0.14 µmol/mg and 0.11 µmol/mg for the SiO_2_ conjugates of 3TC-TP and ddCTP, respectively.

The antiviral activity against HIV-1 subtype A and the cytotoxicity of these formulations were compared to their unconjugated γ-alkynyl triphosphate and parent nucleoside forms. The IC_50_ for 3TC, L4-3TC-TP, and SiO_2_-3TC-TP were 1.7 µM, 5.3 µM, and 0.17 µM, respectively. This would indicate that the conjugation of the γ-alkynyl 3TC-TP to SiO_2_ nanoparticles improved efficacy. However, the IC_50_ of the ddC, ddCTP, L4-ddCTP, and SiO_2_-ddCTP was 4.3 µM, 4.0 µM, 0.86 µM, and 0.96 µM, respectively. This suggests that the attachment of the 4-(prop-2-yn-1-yloxy)butylamine linker (L4) to the c-phosphate of a tri-phosphorylated ddC nucleoside enhanced anti-HIV-1 activity. As the IC_50_ of L4-ddCTP was slightly improved upon that of SiO_2_-ddCTP, it is challenging to conclude the effectiveness of the SiO_2_ nanoparticles for this formulation (106). Similar chemical coupling strategies have been effectively utilized for other inorganic carriers, including gold. TFV-linked gold (Au) nanoparticles had a 0.067 µg/mL IC_50_ against HIV-1 reverse transcriptase in an *in vitro* assay, 15 times more potent than free TFV (107).

Inorganic Au and SiO_2_ nanoparticles exhibit size-, dose-, and coating-dependent toxicity *in vitro* and *in vivo* (108, 109). Smaller particles (1–19 nm) can induce cytotoxicity through oxidative stress, reactive oxygen species generation, DNA damage, and apoptosis (110–113). *In vivo*, Au nanoparticles may accumulate in the liver, spleen, kidneys, and brain, causing organ toxicity. SiO_2_ nanoparticles can induce multi-organ damage and pulmonary inflammation (110–113). The variable acute toxicity of both nanomaterials poses potential health risks and requires careful evaluation for biomedical applications.

## LIPID CALCIUM PARTICLES

As a hybrid inorganic nanoparticle, lipid calcium particles (LCPs) exploit the electrostatic interaction that forms between negatively charged phosphate groups of nucleotides and the positive charge of calcium ions to form a calcium phosphate core (114). This core dissolves under acidic endosomal conditions, generating osmotic swelling and allowing rapid endosomal escape. Adding lipids such as DOPA and DSPE-PEG 2000 can prevent aggregation and allow the addition of targeting ligands. LCPs have demonstrated considerable promise in the oncology field, particularly as nucleic acid vaccine carriers (115–117). Several studies have encapsulated nucleosides/tides, including the antiviral acyclovir monophosphate (ACVMP), albeit for use as an antitumor treatment (118–120). Using a water-in-oil emulsion method, ACVMP encapsulation and LCP size were approximately 70% and 20–30 nm, respectively. Furthermore, *in vitro* and *in vivo* murine models showed enhanced antitumor effect, demonstrating the effectiveness of the LCP platform (118, 119). Interestingly, regardless of continued progress for this platform, limited examples exist for its use as a small antiviral molecule carrier (115–117). This may be due to the complex role Ca^2+^ has in the viral life cycle. Moderate increases in cytosolic Ca^2+^ can trigger Ca^2+^ activation of transcription factors that promote viral replication. Additionally, Ca^2+^ plays a critical role in regulating apoptotic cell death (121). Future work should look to clarify the viability of this platform for the acute treatment of viral infection.

A summary of the nanoparticles discussed in this review is shown in Table 1.

**TABLE 1:** Summary of nanoparticles used for antiviral nucleotide analog delivery

Carrier	Formulation method	Cargo	Size and zeta potential	EE, DL, or other	EE and DL method	Target virus	Admin route^*a*^	Key *in vitro* and/or *in vivo* data	Ref.
Liposome	Thin film extrusion	ddCTP	215 nm	EE = 9%	Direct measurement via liquid scintillation counting	HIV	n/d^*b*^	*In vitro*: 90% inhibitory dose (ID_90_) ≥ 62.5 nM for ddCTP liposomes in cultured human monocyte-macrophages. The ddC nucleoside was 4–5 times more potent.	66
Liposome	Thin film extrusion	ddCTP	300 nm	EE = 26%	Direct measurement via HPLC following MeOH disruption	HIV	Intraperitoneal	*In vivo*: intraperitoneal administration of ddCTP liposomes in mice reduced surrogate HIV viral DNA in MPS cells.	64
Liposome	Thin film hydration and sonication	RDV	114–128 nm, ~−7 mV	EE = 89% (maximum) DL = 11% (maximum)	Direct measurement via UV-spectrophotometry following MeOH disruption	SARS-CoV-2	Inhaled	*In vivo*: inhaled administration of RDV liposome in mice resulted in a ~100-fold increase of RDV-TP in lung tissue compared to intravenous administration. Inhalation significantly decreased RDV-TP in the heart, spleen, brain, and testis.	68
Liposome	Ethanol injection and extrusion	RDV	118 nm, −2 mV	EE = 57%–59%	Direct measurement via UV-spectrophotometry following ethanol disruption	SARS-CoV-2	Intranasal	*In vitro*: EC_50_ = 0.9 µM for RDV liposomes and 0.3 µM for free RDV against SARS-CoV-2 in Vero E6 cells. RDV liposomes were less cytotoxic in Vero E6 and Calu-3 cells. *In vivo*: intranasal administration of liposome-RDV in transgenic mice improved survival rate and reduced SARS-CoV-2 titers in lung and brain.	69
Liposome	Thin film hydration and sonication	RDV	71 nm, −32 mV	EE = 100%	Indirect measurement of supernatant via HPLC	SARS-CoV-2	Inhaled	*In vitro:* RDV liposomes had limited toxicity in A549 lung carcinoma cells.	70
SLN	Phase-inversion technique and layer-by-layer deposition	TFV	153 nm, −51 mV	EE = 8% DL < 0.1%	Indirect measurement of supernatant via UV-spectrophotometry	HIV	Intravaginal	*In vitro*, blank, unfunctionalized, and functionalized SLNs were non-toxic to VK2/E6E7 vaginal epithelial cells across the tested concentration range.	74
SLN	Solvent diffusion	ADV	389 nm, −31 mV	EE = 15% DL = 3%	Indirect measurement of supernatant by UV-spectrophotometry	HBV	n/d^*b*^	*In vitro*: ADV-SLNs significantly reduced 2-day and 5-day HBV DNA in HepG2.2.15 cells at 0.625, 2.5, and 10 µg/mL.	76
SLN	Melt emulsification and sonication	TAF	126–274 nm, −18 to −27 mV	EE = 96% (maximum)	Indirect measurement of supernatant by UV-spectrophotometry	HIV/HBV	Transdermal	*In vivo*: TAF-SLN gel exhibited no significant acute dermal toxicity and limited skin irritation in rats.	78
NLC	Melt emulsification and sonication	RDV	143 nm, −7 mV	EE = 100%	Indirect measurement of supernatant by UPLC-MS/MS	SARS-CoV-2	Intravenous	*In vitro*: EC_50_ = 0.2 µM for NLC-RDV and 1.6 µM for free RDV against SARS-CoV-2 in Vero E6 cells. Enhanced potency against Alpha, Beta, and Delta SARS-CoV-2 variants. *In vivo*: intravenous administration of NLC-RDV in rats resulted in enhanced plasma concentrations of RDV nucleoside metabolite for 12 h.	29
PLGA	Emulsification-solvent-evaporation	TDF	218 nm, −5 mV	EE = 57%	Indirect measurement of supernatant by HPLC	HIV	Oral	*In vivo*: PLGA-TDF nanoparticles showed enhanced oral absorption and prolonged residence time in rats.	83
PBAE liposome	Water-in-oil reverse microemulsion	RDV-TP, MTP	~170 nm, ~−13 mV	EE = 40%–60% DL > 50%	Undefined HPLC method	SARS-CoV-2, influenza virus	Intravenous	*In vitro*: reduced viral titers (TCID_50_) of PRRSV and bovine enterovirus. *In vivo*: intravenous administration of MTP-loaded nanoparticles enhanced pulmonary drug accumulation in H1N1 influenza virus-infected mice.	84
Nanocapsule	Water-in-oil microemulsion	AZT-TP, CDV	~170 nm, −8 to −10 mV	EE ≤ 4% DL = ~2%	Direct measurement via liquid scintillation counting	HIV	Intravenous	n/d^*b*^	88
Nanocapsule with PEI	Water-in-oil microemulsion	AZT-TP	249 nm, −15 mV	EE = 90% (maximum)	Direct measurement via liquid scintillation counting	HIV	n/d^*b*^	*In vitro*: AZT-TP-loaded polymeric nanocapsules resulted in a 10–30-fold higher uptake of AZT-TP by murine macrophages.	89
Chitosan nanoparticle	Ionic gelation	SOF	137 nm, 29 mV	EE = 80% DL = 6%	Direct measurement of SOF inside the chitosan nanoparticle via UV-spectrophotometry	HCV	n/d^*b*^	*In vitro*: SOF-loaded chitosan nanoparticles inhibited HCV RNA at 100 µM. Only one concentration was trialed.	93
Cyclodextrin-chitosan nanoparticle	Ionic gelation and self-assembly	SOF	Cs: 215 nm, 44 mV βCD-SOF:~1.4µm, −22 mV	Loading efficiency = 95%	Unsuitable indirect method measuring UV-vis spectroscopy changes associated with binding	HCV	n/d^*b*^	*In vitro*: cytotoxicity trialed in three cell lines. SOF concentration was not accurately defined.	101
Hyperbranched dendritic nanocarrier	Oil-in-water emulsion and solvent evaporation	RDV	185 nm, −7 mV	EE = 82% DL = 14%	Direct measurement via HPLC following MeOH/DMSO disruption	SARS-CoV-2	Inhaled	*In vitro*: CC_50_ of RDV-loaded dendrimers was comparable to that of the free drug in MRC-5 lung diploid fibroblasts and NR8383 alveolar macrophage cells.	103
SiO_2_ nanoparticle	CuAAC click chemistry	3TC-TP, ddCTP	~146 nm, −9 mV (3TC-TP), −6 mV (ddCTP)	Drug concentration = 0.14 µmol/mg (3TC-TP), 0.11 µmol/mg (ddCTP)	Direct measurement via UV-spectrophotometry following dissolution in NaOH	HIV	n/d^*b*^	*In vitro*: IC_50_ = 0.17 µM (SiO_2_-3TC-TP) and 0.96 µM (SiO_2_-ddCTP) against HIV-1 in MT4 human T lymphoblastoid cells.	106
Au nanoparticle	Citrate reduction and chemical conjugation	TFV	38 nm, −47 mV	Surface weight loading = 16% TFV	Direct measurement via UV-spectrophotometry and thermogravimetric analysis	HIV	Intravenous	*In vitro*: IC_50_ = 0.067 µg/mL for TFV-loaded Au nanoparticles against HIV-1 reverse transcriptase in an *in vitro* assay, 15 times more potent than free TFV. TFV-loaded nanoparticles also had improved antiviral potency against multiple HIV-1 strains in HeLa-derived TZM-bl HIV reporter cells and peripheral blood mononuclear cells. *In vivo*: intravenous administration of TFV-loaded Au nanoparticles resulted in rapid accumulation of TFV in the lungs and liver.	107
LCP	Water-in-oil microemulsion	ACVMP	20–30 nm, 28 mV	EE = ~70%	Direct measurement via UV-spectrophotometry following dissolution in acid	n/a^*c*^	Intravenous	*In vivo*: intravenous administration of ACVMP-loaded nanoparticles significantly inhibited tumor growth in H460 tumor-bearing nude mice.	119

^*a*^ Administration route.

^*b*^ Not determined (n/d) or reported in the cited publication.

^*c*^ Not applicable; ACVMP was evaluated for anticancer activity.

## TRANSLATIONAL CHALLENGES

### Analytical validation

Despite continued innovation in the space, the translation of these antiviral nucleotide nanoparticles has been limited. Formulation methods can be complex multi-day processes, often requiring large amounts of drug, and may potentially lead to degradation or loss of the nucleotide analog. During techniques such as hot-melt and water-in-oil solvent evaporation, the analog stability during heating, solvent addition, and removal should be explicitly considered (122, 123). For example, RDV has been reported to undergo 10% degradation following a 5 h 100°C thermal exposure, illustrating that some nucleotide analogs are not chemically inert under heat stress (124). More broadly, NTPs are intrinsically less stable due to the increased liability of phosphoanhydride bonds, making them more susceptible to hydrolysis and thermal decomposition (125, 126). When the chemical integrity of the nucleotide analogs is not validated after formulation, it may conflate true EE and DL with degradation or process-related loss.

Variable measurement and reporting of EE and DL remain a major challenge, particularly when an indirect measure of unencapsulated nucleotides by UV-spectroscopy is the only evidence provided. For traditional small molecule development and nucleic acid-loaded LNP, researchers often follow analytical methodology set out in accordance with the International Council for Harmonization guideline (127, 128). It is important that the analytical procedure for antiviral nucleotide nanoparticles provides proof of adherence to such guidelines. Often considered the gold standard for quantifying EE, HPLC can be challenging, particularly for some of the discussed formulations due to the constituents used in the nanoparticle and/or the solvents used during formulation being unsuitable for some HPLC columns (129). Fast and simple methods utilizing fluorescent dyes such as RiboGreen are used to directly measure nucleic acid content inside LNP for oligonucleotides such as RNA and DNA (130). However, RiboGreen nor other readily available dyes bind mononucleotides (131). Fluorescent styrylpyridine-based cyclophanes have shown promise for the selective detection of pyrimidine nucleotides such as thymidine triphosphate and cytidine monophosphate (132). Continued development of a simple fluorescent or colorimetric measure of mononucleotides in the context of nanoparticles would be beneficial.

### Preclinical validation

Comprehensive antiviral and cytotoxicity evaluation is crucial for antiviral nucleotide studies. Physicochemical characterization without direct antiviral testing or with incomplete comparisons against the free drug forms limits the assessment of drug efficacy. Additionally, accurate *in vitro* analysis depends on reliable drug quantification. When used to estimate dosing concentrations, inaccuracies in EE may distort effective nucleotide analog exposure, reducing apparent cytotoxicity, while antiviral efficacy may appear diminished. When coupled, toxicity and antiviral data should reinforce prior nanoparticle analysis. We also note that publication of known nanoparticle carriers applied to new drugs is sometimes discouraged, which may unintentionally limit refinement and standardization of the analytical and preclinical framework. More extensive collaboration between virologists, chemists, and formulation scientists will enable the generation of cohesive data sets that meet the analytical and biological expectations of each respective discipline.

**Fig 7:**
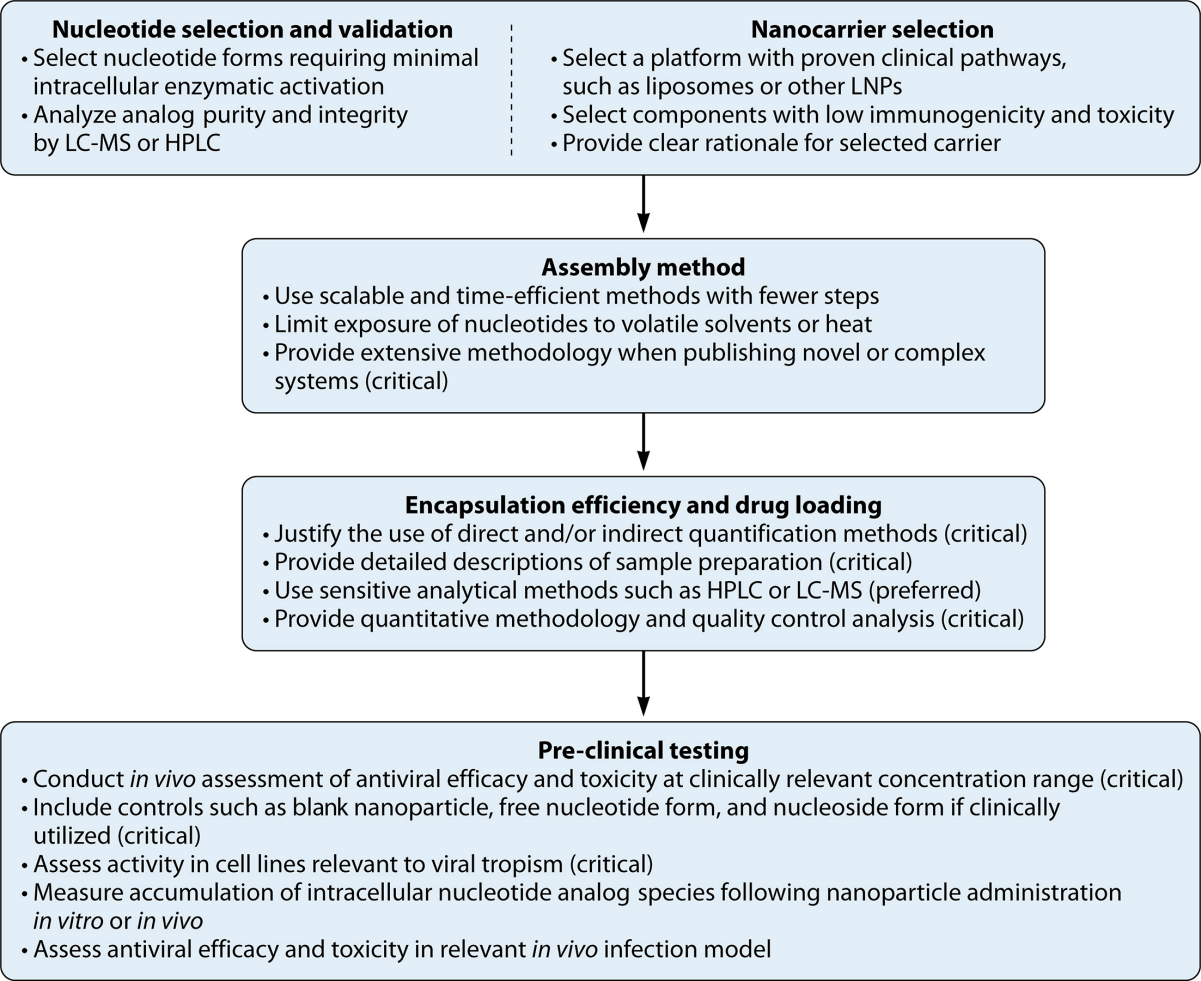
General framework for the development and preclinical evaluation of antiviral nucleotide nanoparticles.

General best practices for the analytical and preclinical evaluation of antiviral nanoparticles are summarized in Fig. 7. The diverse and complex nature of some of the discussed systems may necessitate alternative analytical approaches. Across all systems, prioritize downstream clinical viability and provide extensive formulation description, analytical methodologies, and quality control. Additionally, *in vitro* and *in vivo* evaluations should be conducted with relevant controls in models reflective of viral tropism.

### Clinical validation

Many of the nanoparticle platforms discussed in this review have been clinically validated for prophylactic vaccines, and for small-molecule drug delivery in non-antiviral settings, particularly in oncology (133–135). Numerous nanoparticle formulations have been approved by the FDA and/or European Medicines Agency (EMA) for use in cancer treatment (134). Importantly, several of these clinically advanced nanomedicines involve nucleoside/tide analog chemotherapeutics (134). For example, liposomal formulations of the cytidine nucleoside analog cytarabine (DepoCyt) have received FDA and EMA approval for treatment of lymphomatous meningitis (136). Additionally, a liposomal formulation of deoxycytidine analog gemcitabine was recently reported to have preliminary positive phase 2a safety (ClinicalTrials.gov identifier: NCT05318573) and efficacy study results, with study completion expected in 2029 (136).

In contrast, there are currently no approved nanoparticle formulations delivering antiviral nucleotide or nucleoside analogs for the treatment of viral infection (137). A small number of antiviral nanoparticle formulations have entered early-stage clinical trial evaluation, including inhaled and long-acting formulations of established antivirals (137, 138). However, these programs have largely stalled after phase 1. Notably, the estimated study completion of a phase 1 safety, tolerability, and pharmacokinetics study of inhaled liposomal RDV (ClinicalTrials.gov identifier: NCT04480333) was expected in early 2021, yet to our knowledge, no results have been disclosed to date (139). The lack of translation for antiviral nucleotide-nanoparticles reflects continued chemical and analytical barriers, with the remaining challenge including execution and standardization.

## CONCLUSION/DISCUSSION

Nucleotide analogs remain a fundamental antiviral treatment, yet issues remain with their delivery and activation. This review provides context and discusses recent nanoparticle advances for the delivery and protection of phosphorylated nucleotide analogs from degradation.

Due to their low molecular weight, hydrophilicity, and water-soluble nature, many nucleotide analogs are poorly retained within lipid carriers such as liposomes, SLNs, and NLCs (140, 141). In contrast, nucleotide analogs incorporating lipophilic masking groups such as phosphoramidate ProTides demonstrate substantially improved encapsulation and antiviral efficacy. However, the requirement for additional chemical modifications adds further complexity to their synthesis and metabolic activation, somewhat reducing the potential benefit of these analogs as cargo.

Hybrid nanostructures utilizing lipid, polymeric, or polysaccharide components within a single particle can improve encapsulation and, theoretically, sustain favorable uptake and release. Furthermore, encapsulation can be enhanced using affinity-driven strategies, such as guanidinium-phosphate and phenylboronic acid-ribose pairing. Intrinsic chemical features of the nucleotide are exploited, rather than solely relying on physical confinement within a carrier matrix. Similar affinity-driven systems are currently being explored for LCP delivery of anticancer nucleotide analogs, whereby calcium and phosphate are being utilized to bind and precipitate nucleotides in a dense core (118).

This field has seen immense innovation, particularly in recent years. However, the emphasis on formulation novelty may outpace the maturation of standardized analytical and *in vitro* evaluation methods, affecting comparison and proof of concept.
